# Unused Hemodialysis Acid Concentrate is Dollars and Dialysate Down the Drain: An Opinion Piece

**DOI:** 10.1177/20543581241308631

**Published:** 2024-12-20

**Authors:** Anukul Ghimire, Karthik K. Tennankore, George Vitale

**Affiliations:** 1 Division of Nephrology, Cumming School of Medicine, University of Calgary, AB, Canada; 2 Division of Nephrology, Department of Medicine, Dalhousie University, Halifax, NS, Canada

**Keywords:** acid concentrate, dialysate, green nephrology, health care waste, hemodialysis

## Background

Reducing health care waste is a priority for medical programs, and nephrology is no exception. Specific to the area of nephrology, there is more attention being placed on dialysis, which has significant negative environmental impacts.^1-3^ Hemodialysis (HD) treatments use substantial amounts of water and power and generate large volumes of discarded plastic material and greenhouse emissions.^1,4,5^ As an example, each dialysis session can produce up to 8 kg of plastic waste, requiring up to 21 USD for waste disposal.^6^ Thus, it is not surprising that the annual carbon footprint per dialysis patient is up to 7 times greater than the estimated carbon footprint for the general population.^1,7^ Given the projected rise in the prevalence of patients needing dialysis globally,^8^ the ecological impact of HD will continue to grow.

The World Health Organization (WHO) has broadly categorized health care waste as hazardous vs non-hazardous waste.^9^ Hazardous waste includes all materials that have come into contact with blood and requires more careful methods of disposal. Non-hazardous waste should be recycled whenever possible, which in some centers reduces the cost associated with waste disposal.^6^ Non-hazardous waste includes components of the dialysate that never encounter the patients’ blood during HD. Dialysate is formed from the mixture of (1) ultrapure water, (2) an acid concentrate that contains a specific electrolyte composition, and (3) a bicarbonate buffer that helps create the final dialysate pH. To date, most studies looking to reduce dialysate waste have focused on reducing the prescribed dialysate flow (Qd) to save water.^10-12^ To our knowledge, the quantity of waste related to discarded acid concentrate and the environmental and economic implications of this have not been previously described.

## Waste Associated With Acid Concentrate

**Figure 1.**
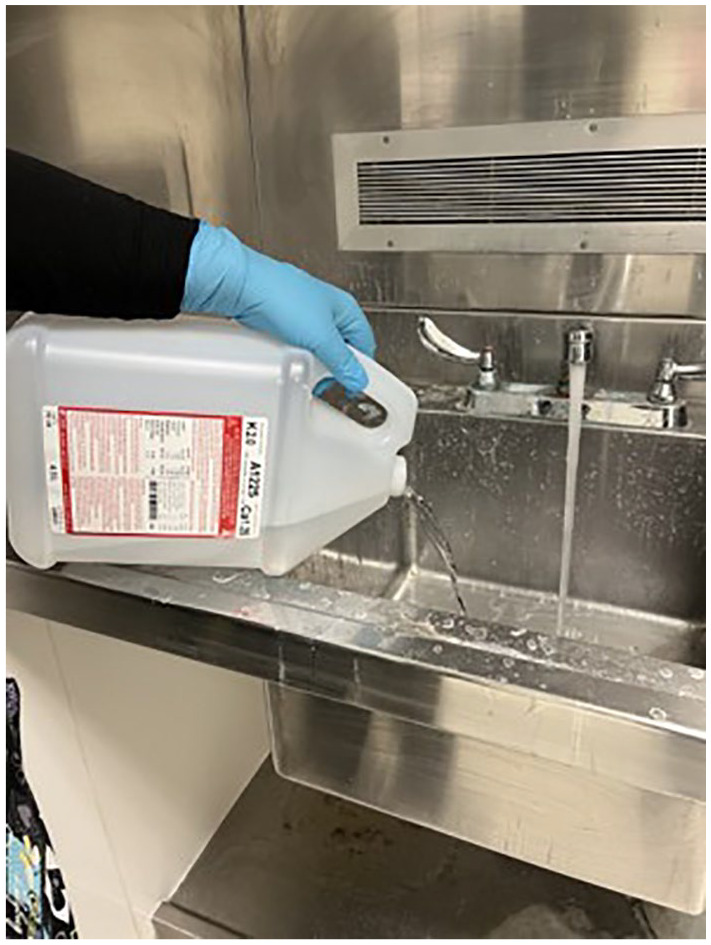
Unused acid concentrate is discarded down the wastewater drain.

In our program, for every 42 mL of ultrapure water used to make dialysate, 1 mL of the purchased acid concentrate^13^ is used. Thus, for a 4-hour session with Qd of 500 mL/min, 2.8 L of acid concentrate is used. If Qd is reduced to 400 cc/min, 2.3 L of acid concentrate is used. In many Canadian centers, the acid concentrate is only available in single-use 4.5 L plastic containers.^14^ As only 2 to 3 L of acid concentrate is used per dialysis treatment, the remaining 1.5 to 2.5 L of unused acid concentrate is ultimately discarded into the wastewater drain system (insert Figure 1). Thus, 33% to 56% of purchased acid concentrate is wasted. This waste is likely greater in programs that use larger acid concentrate containers.^15,16^ This discarded acid concentrate ultimately mixes with the wastewater from the facility and so is thought to be diluted out; formal studies evaluating the environmental impact of discarded acid concentrate are missing. Furthermore, it is unclear whether there needs to be pH monitoring in the drainage systems to evaluate the environmental impact of this waste.

**Figure 2.**
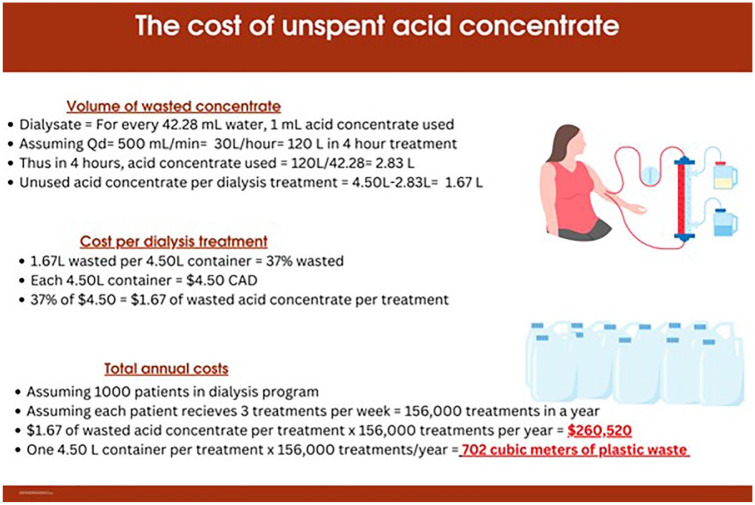
Cost of wasted acid concentrate. Estimated costs are based on cost per purchased container of acid concentrate in the Alberta Kidney Care South program.

**Figure 3.**
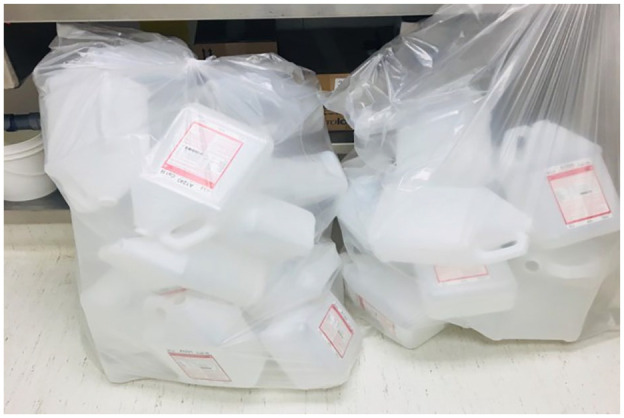
It is estimated that at least 702 m^3^ of plastic waste is produced annually from the plastic containers that store the acid concentrate.

In the Alberta Kidney Care South (AKC-S) renal program with (1) approximately 1000 in-center HD patients being treated with HD 3 times per week, (2) more than 33% of acid concentrate being wasted, and (3) acid concentrates costing $4.50 CAD per 4.5 L container, we estimate that approximately 260 520 L (equivalent to $260 520) of purchased acid concentrate is discarded annually in our program alone (insert Figure 2). Furthermore, we estimate that over 156 000 plastic containers measuring 4.5 L (used to store the acid concentrate) are discarded every year, resulting in 702 m^3^ of landfill waste annually (insert Figure 3). This problem is not exclusive to Alberta; British Columbia also uses 4.5 L acid concentrate solutions for HD^14^ and programs in Manitoba use 5 L containers.^15^ More likely than not, most dialysis programs in Canada are spending a considerable amount of health care dollars on wasted acid concentrate. Although the cumulative financial cost and total production of plastic containers across Canadian HD programs have not yet been characterized, these numbers would likely be alarmingly high. Furthermore, the wasted money and environmental impact will continue to increase as the prevalence of kidney failure treated with HD in Canada rises.^17^

## Solutions to Reduce Acid Concentrate Waste

Strategies to conserve acid concentrate can reduce medical waste and save health care dollars. In the AKC-S program, there is an institutional policy that restricts the use of an acid concentrate container (4.5 L) to only 1 treatment and does not allow the unused acid concentrate to be utilized for another patient’s treatment. This is due to a theoretical risk of transmitting infections between patients. This supposed risk, however, is not supported by evidence. To the best of our knowledge, there have not been any reported cases of infection transmitted through dialysate. It also seems unlikely that most transmissible organisms would survive in the highly acidic (pH = 1-2) nature of the acid solution. Furthermore, the unspent acid concentrate does not come into contact with the patient’s blood, and so there should not be a risk of transmitting bloodstream infections. Thus, it seems logical that using the remainder of an acid concentrate for another treatment is safe and can help reduce wasted solutions. We are not aware of any units in Canada that currently use leftover acid concentrate.

Another potential solution is to use central delivery of acid concentrate. Instead of single-use acid containers, the central delivery allows for bulk use of an acid concentrate and delivers only the amount of concentrate needed for each HD treatment, thereby reducing waste. Here, HD units would receive the acid concentrate in a bulk load, and this would then be pumped into holding tanks which can act as reservoirs. A piped loop system would then distribute the concentrate to dialysis machines.^16^ In a report from the United Kingdom, the installation of a central acid delivery system resulted in annual savings of £22 900 and was estimated to reduce carbon dioxide emission by 16.03 tons.^16^ In Italy, the use of a central acid delivery system led to a substantial reduction in both wasted acid concentrate (351 L as opposed to 12 100 L) and wasted plastic materials.^18^ The major limitation of adopting a central acid delivery system is the upfront cost required to change infrastructure (large-volume drums to store the acid, change in building piping to accommodate the system, etc). In 1 case study, this cost was soon recovered; in the United Kingdom, an initial upfront investment of £43 900 for the change in infrastructure resulted in a 163% return on investment at 5 years for 1 dialysis unit.^16^

Given the notable discordance between the acid concentrate used per dialysis treatment (2-3 L) and the size of the available single-use container (4.5-5 L), it would make sense to reduce the size of the acid concentrate container. In Australia and New Zealand, there are multiple sizes of acid concentrate available for purchase including 3.5 L containers and the use of smaller volume acid concentrate containers can substantially reduce wasted solutions.^19^ Although Health Canada regulates acid concentrates as pharmaceutical products, they have not placed any restrictions on the size of these containers; the chosen volume of 4.5 L appears to be a decision of the vendors. It is not clear why HD vendors in Canada only offer larger-sized containers. Further collaboration between the Canadian Nephrology community and HD vendors is needed to develop more optimally sized acid concentrate containers.

## Local Experience and Next Steps

In the AKC-S renal program, we are working with members of Calgary zone infection prevention and control (IPC) team to develop a protocol to safely conserve acid concentrate. We had proposed a longitudinal project, based on quality improvement methodology,^20^ to use leftover acid concentrate between dialysis treatments. Our outcome measures were to include changes in costs of acid concentrate purchased and the number of plastic containers used per patient undergoing HD treatments in our program. In order to demonstrate safety, we planned to monitor changes in (1) the incidence of bloodstream infections (through monitoring of a pre-existing database that records this information), (2) assessment of concerns relating to workplace safety (eg, spilled acid concentrate) by surveying nurses, and (3) monitoring for any medical errors (eg, using the wrong acid concentrate solution for a dialysis treatment) through established reporting and learning systems that already exist.

Our proposed project consisted of 4 key steps. First, after an acid concentrate is opened, the lid for the container will be retained (instead of thrown out). Second, once an HD treatment is finished, the nurse will wipe the lid using an alcohol-based cleansing swab before closing the container. Third, this opened container will be stored alongside other opened containers (and separated from the unused containers) and will be used for subsequent HD treatments. Fourth, as the opened containers will have a lower starting volume of acid concentrate and will likely run out before the treatment is finished (ie, if the container only contains 1-2 L of the remaining acid concentrate from the previous treatment), the nurse will switch out the empty container for a new container mid-dialysis session. This step does not deviate from current practice, as this is already being done, as an example, when a nephrologist changes the potassium concentration during an HD treatment. Thus, the nurses already have experience changing the acid-concentrate containers during the treatment session. For all of these steps, skin protection (gloves) would be required.

These steps were developed with input from members of the IPC team. Furthermore, from review of policy, our IPC team did not find any consensus document or specific data recommending that unused acid concentrate be discarded. The main limitation to the re-use of the acid concentrate—and implementation of our project—is the manufacturer’s instructions for use (MIFU). The MIFU notes that leftover product should be disposed of immediately after use. This information, however, is not explicitly labeled in the supplier safety data sheet.^13^ Although adherence to the MIFU is a requirement as per Health Canada, the reason for this restriction from the manufacturer is not clear. There is a hypothetical risk of transmitting an infection from the environment; however, our team noted that (1) the process of setting up the acid concentrates is not sterile to begin with and the risk of environmental transmission is always present (even in current practice) and (2) as mentioned previously, infection seems unlikely to be transmitted via this acidic solution. However, without written approval from the manufacturer, we were not able to move forward with our proposed quality improvement initiative to assess the feasibility of using leftover acid concentrate.

Another strategy to conserve acid concentrate involves decanting acid concentrate into empty containers for use. As an example, a total of 9 L of acid concentrate (2 × 4.5 L containers) can be divided into 3 separate 3 L containers by decanting 1.5 from each of the 2 unused 4.5 L containers into a separate, empty container. This practice would be feasible from IPC perspective and would be compatible with the MIFU, as the containers being decanted would not have been used yet and thus do not require immediate disposal (unlike the partially used containers). The process, however, would require additional empty containers (potentially resulting in more plastic waste) and would add additional steps in the disposal of acid concentrate. Currently, leftover acid concentrate is disposed of in a dedicated sink in the HD unit that contains specialized piping to limit corrosive damage from the acid. Health care staff are expected to wear skin and eye protection (gloves and face shield) and respiratory protection (mask and proper ventilation) during this process. Although water is used in this process to rinse the acid concentrate down the drain (as shown in Figure 1), there is no specific protocol for how much water is to be used. Adopting a protocol to decant acid concentrates would require more time from health care staff, expose them to more potential spill hazards from decanting, and would require approval from the workplace health and safety board.

Thus, developing a protocol for the salvage of unused acid concentrate has had many operational and logistical challenges. In our opinion, reducing HD acid concentrate use would be best achieved using smaller acid concentrate containers that are closely matched to a required concentrate volume for a standard HD treatment. Furthermore, moving to a central delivery of dialysate may not be practical for every dialysis center in Canada given the need for infrastructure changes. The use of smaller, more practically sized acid concentrates may be the most feasible long-term change for many Canadian dialysis centers. Unfortunately, this would require changes to the operational infrastructure for HD vendors, which comes with financial costs. Furthermore, it is unclear if the development of smaller-sized containers is a profitable initiative, and so the financial incentive for vendors to adopt this change may be missing.

We believe that the use of more optimally sized concentrate containers should be a focus for advocacy in the nephrology community. Reducing acid concentrate waste can save health care dollars immediately and in the long-term and reduce the carbon footprint associated with dialysis treatments. Reduction in plastic waste supports the goals of the Canadian Medical Association and WHO to develop environmentally sustainable initiatives.^21,22^ Together, Canadian dialysis programs and policy makers will need consider how to leverage networks and resources in order to push private HD vendors to produce small-sized containers. In this process, the first step will be to collect data across the country to quantify the magnitude of this existing and under-recognized problem. The second step should involve dissemination of the data to policymakers and HD vendors. The third step will entail working with the policymakers and vendors to make sensible changes. Our efforts to implement a seemingly simple intervention also highlight larger challenges in health care systems: established procedures without little regard for protecting the environment, institutional restrictions based on minimal evidence, and embedded practice patterns that are difficult to change.

## Conclusion

In summary, we highlight the issue of wasted acid concentrate in Canada and explore the economic and environmental implications of this practice. Utilizing unspent acid concentrate for HD treatments, creating central delivery systems, and developing smaller containers are potential solutions to reduce wasted acid concentrate. We estimate that this wasteful practice costs at least $260 000 annually and produces over 702 m^3^ plastic containers every year in Southern Alberta alone. Further work is needed to understand the cumulative amount of wasted money and acid concentrate in Canada and the associated carbon footprint. Future projects looking at reducing waste by safely utilizing leftover acid concentrate between treatments will need to consider regulations from their local IPC team, Health Canada, and MIFU. In Canada, the use of 4.5 to 5 L containers seems to be standard, suggesting that there is a substantial amount of money being wasted on discarded acid concentrate. Unity among Canadian HD programs and collaboration with both policymakers and vendors is needed to create impactful changes in our practice and to reduce the amount of “dollars and dialysate” being wasted.
